# Iterative editing of multiple genes using CRISPR/Cas9 in *C. elegans*

**DOI:** 10.17912/micropub.biology.000898

**Published:** 2023-11-14

**Authors:** Longjun Pu, Lars Nilsson, Changchun Chen, Jing Wang

**Affiliations:** 1 Department of Molecular Biology, Umeå University, Umeå, Sweden; 2 Umeå Centre for Molecular Medicine, Umeå University, Umeå, Sweden; 3 Wallenberg Centre for Molecular Medicine, Umeå University, Umeå, Sweden

## Abstract

Certain sets of genes are derived from gene duplication and share substantial sequence similarity in *C. elegans* , presenting a significant challenge in determining the specific roles of each gene and their collective impact on cellular processes. Here, we show that a collection of genes can be disrupted in a single animal via multiple rounds of CRISPR/Cas9 mediated genome editing. We found that up to three genes can be simultaneously disrupted in a single editing event with high efficiency. Our approach offers an opportunity to explore the genetic interaction and molecular underpinning of gene clusters with redundant function.

**Figure 1. CRISPR-based method for multiple gene disruption in C. elegans:**
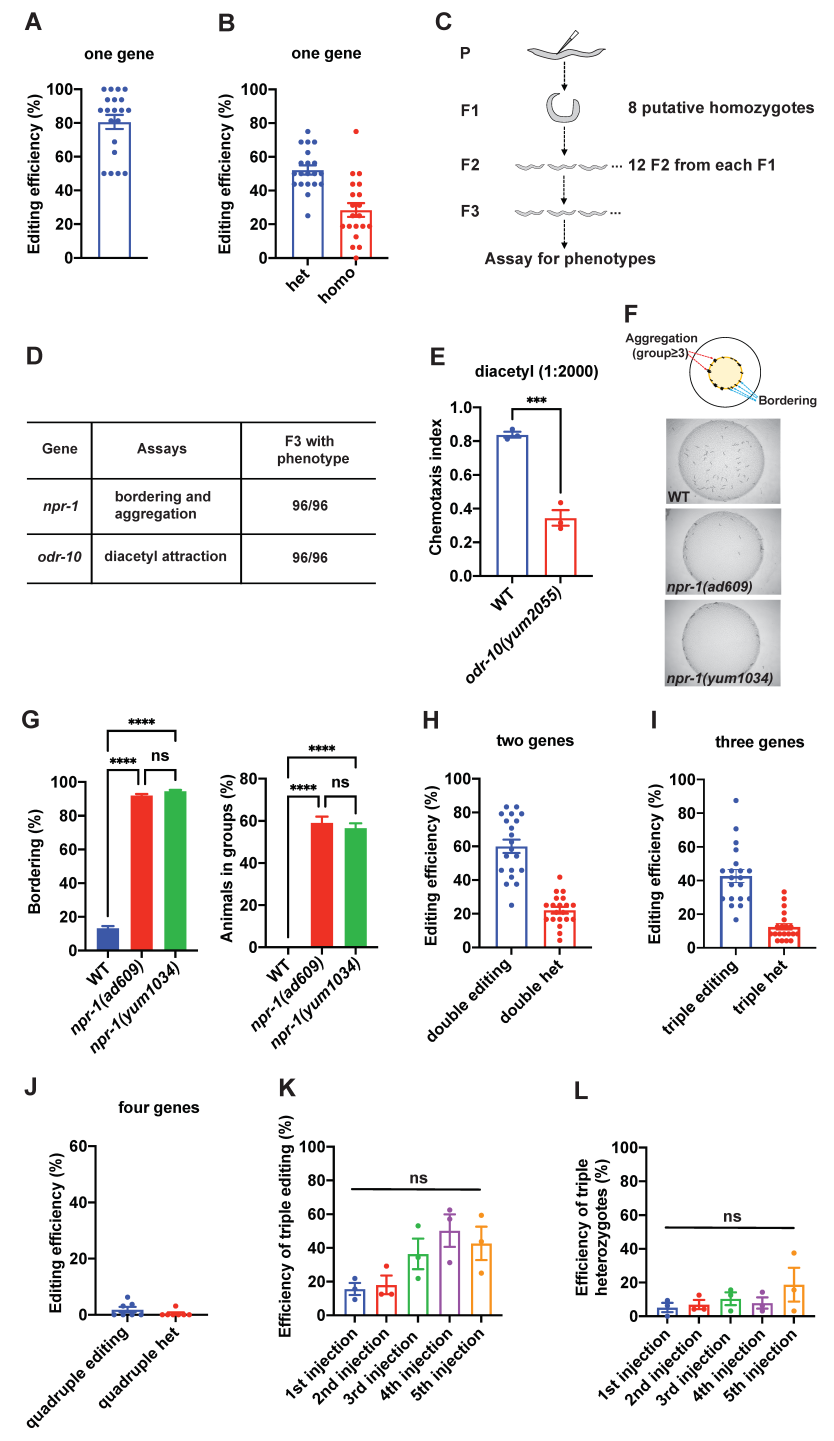
**(A)** The average editing efficiency when one gene was targeted across 20 independent trials. One dot in the plot represents editing efficiency of one independent gene editing experiment. In this and the following figure panels, error bars indicate standard error of the mean (SEM). **(B)** The efficiency of obtaining F1 animals that were either heterozygous (het) or putatively homozygous (homo) of the edited gene based on PCR-based genotyping. **(C)** Strategy of examining if the putative F1 homozygotes were potentially null. **(D)** Two genes *npr-1* and *odr-10* were independently targeted. 8 putative homozygous F1 animals were kept for further analysis. 12 F2 offspring were picked from each F1 homozygotes, and F3 animals were assayed for aggregation ( *npr-1* ) or the response to 1:2000 diluted volatile odor diacetyl ( *odr-10* ). **(E)** Chemotaxis index to 1:2000 diluted diacetyl of animals with indicated genotypes WT (N2) and *odr-10(yum2055)* . *** = *p* < 0.001. *t* test. **(F)** Representative images of bordering and aggregation phenotypes with indicated genotypes WT (N2), *npr-1(ad609)* , and *npr-1(yum1034)* . **(G)** Bordering and aggregation phenotypes with indicated genotypes WT (N2), *npr-1(ad609)* , and *npr-1(yum1034)* . n=5 assays. ****, *p* <0.001; ns = not significant. ANOVA with Tukey’s correction. **(H)** The frequency of obtaining F1 animals that contain editing events in both genes (double editing) and the frequency of obtaining F1 animals that were heterozygous for both genes (double het) when two genes were simultaneously targeted. **(I)** The frequency of obtaining F1 animals that contain editing events in all three genes (triple editing) and the frequency of obtaining F1 animals that were heterozygous for all three genes (triple het) when three genes were simultaneously targeted. **(J)** The frequency of obtaining F1 animals that contain editing events in all four genes (quadruple editing) and the frequency of obtaining F1 animals that were heterozygous for all four genes (quadruple het) when four genes were simultaneously targeted. **(K)** The efficiency of obtaining F1 animals that contain editing events in all three genes (triple editing) in five consecutive rounds of genome editing. ns = not significant. ANOVA with Tukey’s correction. **(L)** The efficiency of obtaining F1 animals that were heterozygous for all three genes (triple heterozygotes) when three genes were simultaneously targeted. ns = not significant. ANOVA with Tukey’s correction.

## Description

Gene duplication and redundancy often pose challenges in attributing phenotypic effects to individual genes and discerning their contributions to biological processes of interest (Ritter, et al., 2013; Ewen-Campen, et al., 2017) . Commonly used approaches such as forward genetic screens often fail to identify the genes with redundant functions. To this end, we explored the possibility of disrupting many genes in a single animal with multiple rounds of CRISPR/Cas9 mediated genome editing. We utilized the previously outlined strategy to disrupt the genes by integrating a single strand DNA oligo (ssODN) via homologous recombination (Dokshin, et al., 2018; Ghanta & Mello, 2020) . To ensure the proper gene disruption, the integration of ssODN involved not only the insertion of in-frame stop codons but also the removal of 14 or 16 bases of coding sequence (Table 2 and 3). It also introduced a unique restriction enzyme cutting site for genotyping. We first sought to determine how many genes could be simultaneously disrupted in a single injection. A collection of GPCR genes were selected for the evaluation (Table 2 and 3). When one gene was targeted, we kept 16 transgenic F1 rollers for the downstream analysis. Overall, the editing efficiency was consistently high across 20 independent trials, exhibiting an average efficiency of 80% ( Figure 1A ). Similar to the earlier observations (Dokshin, et al., 2018) , we obtained both F1 heterozygotes and putative F1 homozygotes ( Figure 1B ). As previously indicated (Dokshin, et al., 2018) , it is likely that certain F1 homozygotes were *trans* -heterozygous, carrying two distinct types of insertions or a combination of an insertion and a deletion that removed the binding site of genotyping primers. Under our experimental conditions, we had an average efficiency of 52% in generating F1 heterozygotes, while the frequency of obtaining F1 homozygotes accounted for 28% in the total of 20 gene editing events ( Figure 1B ). To evaluated if the gene function was eliminated in the putative F1 homozygotes, we targeted at two genes *npr-1* and *odr-10* since their null mutants exhibit clear and robust phenotypes (de Bono & Bargmann, 1998; Sengupta, et al., 1996) . In each gene disruption, we retained 8 putative F1 homozygotes, and singled 12 F2s from each F1 homozygotes. Aggregation and chemotaxis assays were performed at F3 stage ( Figure 1C ). In both cases, no F3 offspring displayed either wild type or heterozygous phenotypes ( Figure 1D -G), suggesting that the putative F1 homozygotes are likely to be null mutants *.* However, opting for F1 heterozygotes is always advantageous in order to maintain a clear genotype of strains, particularly in cases where precise genome editing is required such as generating point mutations or inserting epitope tags (Dokshin, et al., 2018) .

When two genes were targeted simultaneously, we preserved 24 F1 rollers for subsequent analysis after each injection. In a total of 20 independent editing events, the efficiency of concurrent editing for both genes remained consistently high, with an average efficiency of 60% ( Figure 1H ). We also successfully recovered F1 animals that were heterozygous for both targeted genes in all our injections, exhibiting an average efficiency of 22% ( Figure 1H ). The simultaneous editing of three genes occurred less frequent but remained achievable, with an average efficiency of 43% ( Figure 1I ). Picking 24 transgenic F1 animals proved sufficient to obtain the triple mutants in each of our attempts ( Figure 1I ). In particularly, F1 animals containing the heterozygous form of all three targeted genes were obtained in all 20 trials, with an average efficiency of 13% ( Figure 1I ). Genome editing efficiency decreased substantially when four genes were simultaneously targeted in a single injection. We hardly recovered any quadruple mutants in all our attempts if less than 32 F1s were picked. In particular, the efficiency of obtaining F1 animals that were heterozygous for all four genes was very low in a total of 7 editing events ( Figure 1J ). Therefore, using our strategy, it is possible to pursue the editing of up to three genes with relatively high efficiency.

Under certain circumstances it is desirable to disrupt more than 3 genes in a single animal, which means that multiple rounds of gene editing are needed. We wondered if the repetitive gene editing would attenuate the efficiency of editing process. To probe this, we performed gene editing repeatedly in the same strain, with three genes targeted in each round of editing. We conducted three independent genome editing experiments in parallel, targeting a total of 45 genes with the aim of disrupting 15 genes in each animal (Table 2 and 3). Again, 24 F1 rollers were retained in each round of injection. In all three independent trials, we successfully achieved simultaneous editing of three genes in each of the five consecutive rounds of injections ( Figure 1K ). Importantly, we did not observe any noticeable reduction of editing efficiency for isolating F1 animals with triple editing or triple heterozygotes throughout the experiments ( Figure 1K and L ). These data suggest that repetitive genome editing in the same strain of *C. elegans* does not significantly affect the editing efficiency. We anticipate that this approach can be used to disrupt the redundant genes or a set of genes within a specific family in *C. elegans* .

## Methods

***C. elegans* maintenance**

*C. elegans* strains were maintained under standard conditions (Brenner, 1974) . The Bristol N2 were used as wild type. Strains used in this study were listed in Table 1.

**CRISPR-based gene editing**

The strategy involved the homology-directed integration of the single strand DNA oligo (ssODN) (Dokshin, et al., 2018) . The optimized ribonucleoprotein complexes containing Cas9 protein (IDT, #1081059), predesigned crRNA (https://eu.idtdna.com/site/order/designtool/index/CRISPR_PREDESIGN) and tracrRNA (IDT, #1072534) were mixed with ssODN donor template (synthesized by IDT) and a roller co-injection marker (pRF4:: *rol-6(su1006)* ) (Mello & Fire, 1995) , and injected into the gonad of *C. elegans* . The predesigned crRNAs targeted at the earliest possible exon, or the common exons if different splicing isoforms exist (Table 3). The *rol-6* marker plasmid was prepared with midi-prep kit (QIAGEN, Cat. No.12143). The ssODN templates contained two 35-base homology arms flanking the targeted PAM sites. Between the homology arms, two in-frame stop codons were included. A unique restriction enzyme cutting site was also built in for genotyping. The insertion of ssODN introduced the stop codons and restriction enzyme sequence into the targeting site while simultaneously generated frameshift. The F1 roller animals were picked and genotyped for the integration of ssODN. Most of the genotyping primers amplified the fragments between 400 bp and 1000 bp surrounding the ssODN insertion sites. Restriction enzyme digestion of PCR products would generate two fragments of different sizes in the homozygous animals, three bands in the heterozygotes and only one band in the wild type. For many genotyping primers, longAMP Taq polymerase (NEB, M0323L) worked much better for the amplification. The injection mixtures for the disruption of different number of genes were prepared as the following:

i) One gene (Dokshin, et al., 2018) :

1) 0.5 μl Cas9 (10 mg/ml from IDT)

2) 5 μl tracrRNA (0.4 mg/ml in IDT duplex buffer)

3) 2.8 μl crRNA (0.4 mg/ml in IDTE pH7.5)

4) Thoroughly mix these three components and incubate the mixture at 37°C for 10 to 15 minutes.

5) 2.2 μl ssODN (1 mg/ml in nuclease free H _2_ O)

6) 2 μl *rol-6* co-injection marker (600 ng/μl in nuclease free H _2_ O).

7) Use nuclease free H _2_ O to bring the volume to 20 μl.

8) Spin at 14 000 rpm for 10 minutes at room temperature, transfer 17 μl of the mixture to a new tube for the injection.

ii) Two genes:

1) 0.5 μl Cas9 (10 mg/ml from IDT)

2) 6 μl tracrRNA (0.4 mg/ml in IDT duplex buffer)

3) 2 μl of each crRNA (0.4 mg/ml in IDTE pH7.5)

4) Thoroughly mix these three components and incubate the mixture at 37°C for 10 to 15 minutes.

5) 2.5 μl of each ssODN (1 mg/ml in nuclease free H _2_ O)

6) 2 μl *rol-6* co-injection marker (600 ng/μl in nuclease free H _2_ O).

7) Use nuclease free H _2_ O to bring the volume to 20 μl.

8) Spin at 14 000 rpm for 10 minutes at room temperature, transfer 17 μl of the mixture to a new tube for the injection.

iii) Three genes:

1) 0.5 μl Cas9 (10 mg/ml from IDT)

2) 6 μl tracrRNA (0.4 mg/ml in IDT duplex buffer)

3) 1.9 μl of each crRNA (0.4 mg/ml in IDTE pH7.5)

4) Thoroughly mix these three components and incubate the mixture at 37°C for 10 to 15 minutes.

5) 2.1 μl of each ssODN (1 mg/ml in nuclease free H _2_ O)

6) 2 μl *rol-6* co-injection marker (600 ng/μl in nuclease free H _2_ O).

7) Spin at 14 000 rpm for 10 minutes at room temperature, transfer 17 μl of the mixture to a new tube for the injection.

iv) Four genes:

1) 0.5 μl Cas9 (10 mg/ml from IDT)

2) 6 μl tracrRNA (0.4 mg/ml in IDT duplex buffer)

3) 1.7 μl of each crRNA (0.4 mg/ml in IDTE pH7.5)

4) Thoroughly mix these three components and incubate the mixture at 37°C for 10 to 15 minutes.

5) 1.9 μl of each ssODN (1 mg/ml in nuclease free H _2_ O)

6) 2 μl *rol-6* co-injection marker (600 ng/μl in nuclease free H _2_ O).

7) Spin at 14 000 rpm for 10 minutes at room temperature, transfer 17 μl of the mixture to a new tube for the injection.

**Behavioral assays**

Aggregation and bordering were assayed as described previously (Laurent, et al., 2015; de Bono & Bargmann, 1998) with minor alterations. L4 animals were picked to a fresh plate 24h before assay. Assay plates were seeded with a 1-cm diameter OP50 lawn two days earlier. Sixty day-one adults were picked to one assay plate, and bordering and aggregation scored 2h later. Chemotaxis assays were performed as previously described (Yoshida, et al., 2012) . Low concentration of diacetyl was prepared by diluting it with pure ethanol (1:2000). 1 μl of diluted diacetyl was placed on two spots at one side of the 9 cm assay plates, and 1 μl of ethanol was added on two spots on the other side. 1 μl of NaN _3_ (1M) was also added to those spots. About 150 synchronized day one adults were used in each assay, and were allowed to roam for 1 hour. The assay plates were stored at 4°C before counting. The chemotaxis indices were calculated as (the number of worms in the attractant area – the number of worms in the control area) / the total number of worms on the plate.

## Reagents

**Table 1.** Strains used in this study

**Table:** 

**Strain**	**Genotype**	**Source**
N2	Wild type	CGC
DA609	*npr-1(ad609) X*	CGC
CHS1173	*odr-10(yum2055) X*	This study
CHS1057	*npr-1(yum1034) X*	This study
CHS1695	*srh-185(yum2493) srh-186(yum2494) srh-187(yum2495) V*	This study
CHS1696	*srh-185(yum2493) srh-186(yum2494) srh-187(yum2495) srh-190(yum2496) srh-192(yum2497) srh-193(yum2498) V*	This study
CHS1697	*srh-195(yum2500) II; srh-185(yum2493) srh-186(yum2494) srh-187(yum2495) srh-190(yum2496) srh-192(yum2497) srh-193(yum2498) srh-194(yum2499) srh-199(yum2501) V*	This study
CHS1698	*srh-195(yum2500) II; srh-185(yum2493) srh-186(yum2494) srh-187(yum2495) srh-190(yum2496) srh-192(yum2497) srh-193(yum2498) srh-194(yum2499) srh-199(yum2501) srh-200(yum2502) srh-201(yum2503) srh-203(yum2504) V*	This study
CHS1699	*srh-195(yum2500) II; srh-185(yum2493) srh-186(yum2494) srh-187(yum2495) srh-190(yum2496) srh-192(yum2497) srh-193(yum2498) srh-194(yum2499) srh-199(yum2501) srh-200(yum2502) srh-201(yum2503) srh-203(yum2504) srh-206(yum2506) srh-207(yum2507) srh-208(yum2508) V*	This study
CHS1700	*srh-166(yum2716) srh-167(yum2717) srh-169(yum2718) V*	This study
CHS1701	*srh-146(yum2719) srh-147(yum2720) srh-148(yum2721) srh-166(yum2716) srh-167(yum2717) srh-169(yum2718) V*	This study
CHS1702	*srh-146(yum2719) srh-147(yum2720) srh-148(yum2721) srh-149(yum2722) srh-154(yum2723) srh-159(yum2724) srh-166(yum2716) srh-167(yum2717) srh-169(yum2718) V*	This study
CHS1703	*srh-146(yum2719) srh-147(yum2720) srh-148(yum2721) srh-149(yum2722) srh-154(yum2723) srh-159(yum2724) srh-166(yum2716) srh-167(yum2717) srh-169(yum2718) srh-174(yum2729) srh-177(yum2730) srh-178(yum2731) V*	This study
CHS1704	*srh-146(yum2719) srh-147(yum2720) srh-148(yum2721) srh-149(yum2722) srh-154(yum2723) srh-159(yum2724) srh-166(yum2716) srh-167(yum2717) srh-169(yum2718) srh-174(yum2729) srh-177(yum2730) srh-178(yum2731) srh-179(yum2732) srh-180(yum2733) srh-183(yum2736) V*	This study
CHS1705	*srh-288(yum2617) srh-289(yum2618) srh-290(yum2619) V*	This study
CHS1706	*srh-288(yum2617) srh-289(yum2618) srh-290(yum2619) srh-291(yum2622) srh-292(yum2623) srh-293(yum2624) V*	This study
CHS1707	*srh-286(yum2620) srh-287(yum2621) srh-288(yum2617) srh-289(yum2618) srh-290(yum2619) srh-291(yum2622) srh-292(yum2623) srh-293(yum2624) srh-295(yum2625) V*	This study
CHS1708	*srh-297(yum2627) II; srh-286(yum2620) srh-287(yum2621) srh-288(yum2617) srh-289(yum2618) srh-290(yum2619) srh-291(yum2622) srh-292(yum2623) srh-293(yum2624) srh-295(yum2625) srh-296(yum2626) srh-300(yum2630) V*	This study
CHS1709	*srh-297(yum2627) II; srh-286(yum2620) srh-287(yum2621) srh-288(yum2617) srh-289(yum2618) srh-290(yum2619) srh-291(yum2622) srh-292(yum2623) srh-293(yum2624) srh-295(yum2625) srh-296(yum2626) srh-298(yum2628) srh-299(yum2629) srh-300(yum2630) srh-304(yum2633) V*	This study

**Table 2.** ssODNs used for genome editing in this study

**Table:** 

Gene	ssODN
srh-185	TCGAGTCACCCTATATTTTACTACCAGCTATGGCTtaagaattctaaTTTTAGACCAGTTTTCGGTCGATTGCCAGGAGCAG
srh-186	CATTGAGTTTATTTATTATTCCATTTATTATGTGGtaaaagctttaaTTCCATTGGGAATTTTCCAATATATTGCTATAAGT
srh-187	TAATTTTATTTGATTACTCTCTTGGAATTCTCACTtaaaagctttaaTACCGTACCTTGCAGGATTTCCGGTCGGGTTACTC
srh-190	TCGATTATTCACTAAATTTTTTATCATGCCCATTTtaaaagctttaaTAGCTGGCTATCCACTTGGAATTTTTAAATACTTC
srh-192	TAAAATACACCAGTATGCCTCTGGACTATCTAACAtaaaagctttaaTTGGTGCCTgtaagttctgaaaaaatatttgttta
srh-193	CAGTTCCGTTTTTGCTCATTCCGAAAGGCGCGGGAtaaaagctttaaCACAATATACAGACGTCCCTTTAGTTTATCAAACA
srh-194	TAGCAGTTCCATTTTTGCTTATTCCGAAAGCTGCGtaaaagctttaaTGTCTAAATATACAGATATTTCTTTGGCATATCAA
srh-195	CGTTGAGCCTACTCACCGCACCGTTTGTCCTGGTTtaatctagataaACCCGCTTGGCTTATCAAAATACACAAATGTTCCG
srh-199	TATCACCTTTTGCTGCGGGCTTTCCACTTGGTCTGtaaaagctttaaTGTCAGTTGTTGCACAGTCAATATATTTTATAATA
srh-200	TTACCATAATGACGATTCCATTTATTTTAGCACCAtaaaagctttaaCACTTGGAGTGCTTAGACTTTTTGGAGTTCCTACA
srh-201	TCACAATACCATTCATTTTGGCTCCAGGACTTGCTtaaaagctttaaTTTACAAGTATTTTAACGTTCCGTTTATGATTCAA
srh-203	CGGGGTTTTCGCTTGGCTTGGCAAAATATCTGAGTtaaaagctttaaCAGCGTTGACAGCGGTCTATTGTTTTGGACgtagg
srh-166	TACCTGCCTGCGCCGTATATCCACTTGGAGTACTAtaagaattctaaTTCAACTGTTTTTCAAGCCTACGTAGGGGTTTCCC
srh-167	CGGTGATTTTATTCTTTGAAGAACGATATCACAAGtaagaattctaaGGTCAAGCGGAAGAAAAAGTTTCTCAAGAAAATGT
srh-169	CAATTCCGGTGTTAACACTACCAATTTGCTCCGGTtaaaagctttaaCAGTAGTCTTAGGTATTCCTACAAACATTCTAACG
srh-146	ATTTTAGTCTGTTAACTATGCCAGTATTGCATTTAtaaggatcctaaATCCGCTCGGTATTCTCTCATTTTTTGGAGTTCCA
srh-147	TGAGAAAATGGTATCGTTTATTATTTGCAACATTAtaaggatcctaaTAACATTTCCCGTTCCCGTATATTTGTCTCTTCCG
srh-148	TATATATGCCAGTCGCGCTGGTACCAGTTTGTGCTtaaggatcctaaTTCTTAAACGATTCGGGGTTCCTAGTTTGGCGCAA
srh-149	CTATGCCAGTTTTACACTTACCTGTTTGCGGAGGCtaaggatcctaaTAGCATTACTTGGAGTTCCAACTTCATTGCAAACC
srh-154	GCTTACTTTCATTTTTTGGGGTTCCAAGCTCGTTGtaaggatcctaaTCTGTTCACTAGCAGgttggttcttaagaatgatg
srh-159	TCATTATGCCAGTGCTACATTTGCCTGTTTGTGGAtaaggatcctaaCTTACTTTCATTTTTCGGCGTTCCAGTCTTATTGC
srh-174	GAGCAATCGTGGATTTTTATCTGAGCTTCATTTCAtaaaagctttaaTACCCGTTTGCTCTGGATATCCATTGGGCTTCTCG
srh-177	TTTTGTTTTTTGAGGATCGACATCATAGACTGGTCtaagaattctaaAGAAGAATTGGAAACGAGTTTTGTATATTTTCAGT
srh-178	CCCTGCTTCTGGGGATCCCAACAAGTGTCCAGGTTtaagaattctaaGTTTGTTGGGGTCATCGGTGTGACTATTATGTTAT
srh-179	GTGCGACTTTGGACGTATTTTTTAGCTTTCTCGCGtaaaagctttaaTGCCCGGTTGCTCGGGGTATCCATTAGGAATCTCT
srh-180	CAATATATACACTTGGATTTGGTCAAGTCATAGGGtaagaattctaaAGGCTTATATTGGGTACAGTGTAGTTGGAGgtaat
srh-183	CTTCTCCCGTACTAAATTTGCCGGCATGTTCTGGAtaagaattctaaTAACGAAACTTGGGGTTCCTACAGCGATTCAGTTG
srh-288	TAATCATGAGTTTCTTTGCTCAGCCATTCCTTTCTtaaaagctttaaTCCCAATGGGAGTTTTGCATTGTATTGGAGTGGAT
srh-289	TCTTTGCCCAACCATTCATCAGTGCTCCGTTTACTtaaaagctttaaTTTTGCATCGTATTGGAGTGGAGACTGACCTTTTA
srh-290	AGCAACCATTTATATGTATGCCTGTTCTAGCAGGAtaaaagctttaaTGAAATGGTTGAACGTGGAGACGGGGGTCATGGTG
srh-291	GCTGGCGCTACACTCGGTATCCATTTTTAACCCTGtaaaagctttaaTACTTGCCTCTACCGCATCATATCTGGAGATCCCA
srh-292	CAGTGAAATGGAGTCTATTTGATGTACACCTATGGtaaaagctttaaTGTTCTTGAGTTTCTTCGTTCAACCATTGGCATTT
srh-293	TGGCTCAGCCATTTTTCTGTACACCGACCATGGCTtaaaagctttaaTTCTGAGTTTAATTGGCGTGCCTAATGATCTTCTG
srh-286	TCGGAATGCTCGAGAATCGTTACTTTCAAATCTTCtaagaattctaaATGGCGGTACTTTCGCTATCCATTTCTTTTTATCA
srh-287	TAGCTGGATTCCCGCTGGGGCTCTGGAGCTGGCTGtaaaagctttaaTCGTGATGTTTCTTGGATTTACCACTGCTTTTTgt
srh-295	GCTATGCTGGTTACCTTTTAGGAATTTTAAACTTTtaagaattctaaATGCTCAAATTTTAGCAATAAGAGCTGTTTTTATG
srh-296	CCTTGGATATATTACTGAGCTTACTTGCTCAACCAtaaaagctttaaGTTTCTAGCAGGATTTCCGTTAGGCATTCTGAAGT
srh-297	TCTTAGACATTTCCATTAGCCTGCTCGCCCAGCCTtaaaagctttaaGGTATTTGCTGGATATCAATTAGGGATTTTGAGCT
srh-300	CCGCTTCCTTGGATTTATCCATAAGCTTGCTTGCTtaatctagataaCACCGGCGTTTGCCGGGTTTTCACTTGGTATTTGG
srh-298	TTTCTCTAGGAGTGCTGAAATGGGTTGGAATACCTtaaaagctttaaTGGTGATCTCGACAATTTTTATGCgtgagttcttg
srh-299	CAATTACTCTATTCATGCAACCGTATTACTGTACTtaaaagctttaaTCTCACTTGGTCTCTGGAGTTGGACAAGTGTTCCC
srh-304	TTTGCTCTCCAGCTTTTGCTGGGTTTCCCCTTGGAtaagaattctaaAAAAGGGATCCCCATGGATGTTTTGGTTGTATGTG
srh-206	TCATGTTCTTCGACAATTCTGTGACACTTTTGGGTtaaaagctttaaCAACTAGGCTGGCCGGATATTCGCTTGGATTATTG
srh-207	CTGTAACAGTGCTAGGTATTCCGTTTGTGTTGGCTtaagaattctaaTTTCACTTGGATTGCTGCAATACTCGAATTACTCA
srh-208	TGATGGCATTAGACTATTCGGTGACTGTAGTGGGTtaaaagctttaaCAACTAGGATAGCTGGGTTTTCGCTCGGATTGTTG

**Table 3.** Genome editing related material used in this study

**Table:** 

Gene	crRNA	Genotyping forward	Genotyping reverse
srh-185	GCTATGGCTGGAACTTCAAT	CGTTTCAACAAAGTCCACTCGGTTCTATC	GTATACCTTGAAAACTGCAAGCACCG
srh-186	ATTATGTGGCCAATTATGGG	ACTAACATTTAGTCATGAA TTCAAGCGCG	ACCATATAGAGAATTGCAGCCGAGTAGT
srh-187	AGGTACGGTAGCAGGAGCAC	AGGCAATTAGAAGTAGCATTAAATTGTGCA	TTACCAAAACTGTCTGACGAAAAATTTCGA
srh-190	ATGCCCATTTATTTTGATAC	TATGTTTTTTCCCGTCACTCGGTTCTAC	TCGAGACTACTGATATTATCATGCCTGGA
srh-192	CTATCTAACAAGTATAGTTA	AATGAACTACTCATGTATTGCAAAAGCCA	AAATTTGCAACCTGTAAATCTACGCACC
srh-193	GTATATTGTGATACACCAAG	TCGGAAACAATAATTGTCAGTTTCCTTCTT	GCTAATAGTAAGGAACATTGGGCGATCAA
srh-194	AAAGCTGCGGGGTATCCACT	GAAAATTAAACTCAAAGGAATAGCGCCAGT	GCTAACTCTAGGAACATGGAAGTCAGTATC
srh-195	GTCCTGGTTAATGAAGGTGC	TTGTTAAACTGCCCCACAAATGGTTTTC	ATGTAGGTTTTCGCAGTACTCCTATAGTG
srh-199	CTTGGTCTGCTTCGTCTCAC	ACCTACTTTTCATCTGCTGACATTATGACG	TTGTAGTGCATGTGTCTGTTCTGGAAC
srh-200	ACTCCAAGTGGAAAACCCGC	ATGAATTTTTCTTGTCATCCTGACGTTGG	GAAGAACTATTACGTGATTTGCCACCAG
srh-201	GGACTTGCTGGGTATTCACT	GTATTCCACAAGAAGATTATTTTGGCTCTCC	TTTGCATTATTGGTGAAGGTTTTTGGAGTT
srh-203	GTCAACGCTGGCACGATAAA	TCTCCTCAATTTCTAGCAATCTCTATGCA	CGGATATTTTTCCTTAGGAATCGGCATC
srh-166	TTGGAGTACTAACGATGCTT	TCAACTTCCCTTTGCTAATTTCACTTGTC	TATACTTAAGATGAAAAATGCGCCCTGTG
srh-167	TCCGCTTGACCTTTGCACGT	AGAAATGTGCACCGAAACTTTCAGTTAC	ATTGACAGAATGAAAAAGCCAGTACGGG
srh-169	AAGACTACTGCGAGCCCAAG	GCAAACCAAAGTTGATTGAATCAGTTTAGC	GAAAGTGGGAGCAACGAATGTTGAAG
srh-146	TTGCATTTACCTATTTGCGG	TATGCACAATTTACTTCAGTTATGTGCTCC	ATGTACAAAGTAAGGTAGACATCGCTTGC
srh-147	TGCAACATTACACTATGCTC	CAACAAACCATCAATCAAAACCGAGCTAG	GGTTTCATACACAGGTACGCTTTTATTTCA
srh-148	GTTTGTGCTGGCTATACACT	CCATAGAACTGTTACTGATAGCACAAGG	CACAATATTCCGACATGTAAAGCTGTGAAA
srh-149	AGTAATGCTAGAACGCCGAG	CCCAGTTCTGATTTCTAAAGTGCACATT	GGTCTTGTGACCGTGAATCAATCGATA
srh-154	AGCTCGTTGCAAGTTTATGT	CAGATGACATAAACTATGCCCATTGTTACC	GAAGCAAAAAGTATATGGGAGCAGGGTA
srh-159	ATGAAAGTAAGCCAAGCGGA	TCTCAAGTTTTTCTCAGTGATAGCTGCTAC	CCCGAATTGCATCAACAGTTGAGATATAAG
srh-174	CAAACGGGTAAAGTGAGCAC	CAGCACCGATCGAAAACATATGTATGAG	GCTATGAAAGTTGCAGAAAACGCGTAAT
srh-177	AGACTGGTCTATAGGTCCAA	GTGTAAAAACCACAATAATAAAGCCCAGCA	CTCCATCTTTCATTAGGAGTATCTGACGG
srh-178	CCCCAACAAACGAGATACCC	TTTTGGCTCCGACACTTTCTACTCC	TCGGTCCAAGCCCGTAAAACTTAG
srh-179	CAACCGGGCAAAGTTAGGAC	CCATGGCATCCTCTCAACATTGACTATG	GCATCACGTAACTCAGAGCAATAGTGTAA
srh-180	ATATAAGCCTGAACTTCTGT	GAAACTGACATTTTCTATGCAACAACTCTT	AACCATTCTTATGGTACAATAGTGGCGG
srh-183	AGTTTCGTTAGAACCCCTAA	TCATGTTATTTATTGAGGAGGCAAATGCG	GTCGGTATAGGAAATGTAAGTGAGATTGTG
srh-288	CCCATTGGGAACCCTACAAA	CATATAGCGTATTGCTTTCGGAACAAGTG	ACATTGGAAGAAACGCGTTGACTACG
srh-289	TCCGTTTACTGGGTTCCCAA	GACACGTAACATAAGAACAAACGGCTAA	AGACAGGCATCACTGAGTTGATAAGGTTA
srh-290	AACCATTTCATCACACCCAT	GAACTCATGGGAGCACCTTGTTTTTG	CGCTCTACCGAAATAGCCCAAATTTTTC
srh-291	TTAACCCTGAACTACCTAAT	AAAGTATCTGGGGCTGCTAACAATATGA	ATACACCATGACTAATGTTGATAGAGCTCC
srh-292	ACACCTATGGTCATCACTAA	TTGTAGGAAAAATCCTTGTCTCGCATTCC	GAATAACGAATGTAGCGCCAGCATGTA
srh-293	ACCATGGCTGCTTTCCCACT	TCCATTTCCTTCATGGTATCCTCTATCATC	GCCAGGATCTGTCCTCATCACTAATAAC
srh-286	AGTACCGCCATTGACTCTGT	CCGGAACCTATTAAAGGGATTTTGTATAACA	CGATAGGGTATAGAACTATTTTCGCATCGC
srh-287	AGCTGGCTGGAAGTGGACAC	AATATTGGTCGATTGGGGTCAACTTGTC	GCGTAACTCTGTTCGGGGATCATAAAATA
srh-295	ATTTGAGCATCCGTAGGCAC	ACTTTAAATTCCTACCGAAATCTTTCTCACA	TCGGATCAATAATACACAACAATTCGATAGAT
srh-296	CTGCTAGAAACGGAGAGCAC	ATTTTCTCCGATTTGTCACTCGATGCTTC	GGGGTAGTGTAGTACTGCTGTAAAATTACT
srh-297	CAGCAAATACCGGAGCACAT	AACAAGAAAAAGCCCATAGTTACTTCCTTC	AATGAGCCAGTGTTCTCTCATTATTTTTCAT
srh-300	AACGCCGGTGTGCACATGAA	TCCCTTTTACATACTGTTAGCAATCAGGTT	ATCATGCAGTATTTTGGCAGGGACTC
srh-298	TGGAATACCTACGGAGGTGC	GTGATCCCCAGGTCTACTCTATTATTTGC	GAAATTTTCAAAGTCGGCCCAAAATAGGC
srh-299	ACCAAGTGAGAGCCCAGCAT	GCAGAGCCGTCGTGTTACATACAATTAG	CAGTAGGCAATTGCAAGAATGTGATTTGC
srh-304	TTCCCCTTGGAATAGTGCAA	TATGCAAGGTTACTTCGTTCAAGCCTC	GGCTAAAGCTTAGATTTAAGCTACGGCT
srh-206	AGCCTAGTTGCCAATATAAA	ATAATCACGACTCCTGCCATAAACTCG	GCATTGGATAACGCATGTCCTCAATTAT
srh-207	GTGTTGGCTACTAAGTTAGC	GCAGTCTCACTTTTTGGATTCAGTTGAC	TTGCGTGAAGGGTCATGTCTTCAAC
srh-208	ATCCTAGTTGCTAGTACATA	GCCTAACTTCAACTACTACGATTCACCTC	GCCCAATCCTACCTTAAAGATATCCTGC
